# Supplementary material for: Prevalence and correlates of physical fighting among adolescents in Paraguay: Findings from the 2017 national school-based health survey
Source: PLoS One. 2022 Dec 30;17(12):e0279402. doi: 10.1371/journal.pone.0279402 (PMC9803110; doi:10.1371/journal.pone.0279402)
Supplement: S1 Table — (DOCX) [file pone.0279402.s001.docx]

**Table 1: Independent variable derivation from Paraguay GSHS survey data 2017.**

| **Survey Question** | |  | **Coding** | |  | **Variable** |
| --- | --- | --- | --- | --- | --- | --- |
|  |  | | | **Individual-level variables** | | |
| How old are you? | |  | 13–18 years, aged less than 11 and 12 coded as aged 13. 18 years and older coded as 18. (coded continuous) | |  | Age |
| What is your sex? | |  | Male (1) Female (0) | |  | Sex |
| During the past 12 months, how often have you been so worried about something that you could not sleep at night? | |  | Most of the time/always (1) Never/rarely/sometimes (0) | |  | Anxiety |
| During the past 12 months, did you make a plan about how you would attempt suicide? | |  | Yes (1) No (0) | |  | Suicide Plan |
| During the past 12 months, how often have you felt lonely? | |  | Most of the time/always (1) Never/rarely/sometimes (0) | |  | Loneliness |
| During the past 30 days, how many days did you miss classes or school without permission? | |  | 0–2 times (0) 3 or more days (1) | |  | Truancy |
| During the past 30 days, on how many days were you bullied? | |  | 0 times (0) 1 or more times (1) | |  | Bullying |
| During the past 7 days, on how many days were you physically active for a total of at least 60 min per day? | |  | 3 days or less (0) 4 days or more (1) | |  | Physical activity |
| How much time do you spend during a *typical* or usual day sitting and watching television, playing computer games, talking with friends, or doing other sitting activities? | |  | 2 h or less (0) 3 h or more (1) | |  | Sedentary |
| How old were you when you had sexual intercourse for the first time? | |  | Never had sex or had after age 14 (0) Had sex at age 14 or earlier (1) | |  | Early sexual debut |
| During the past 30 days, on how many days did you have at least one drink containing alcohol? | |  | 0 days (0) 1 or more days (1) | |  | Alcohol use |
| During the past 12 months, how many times were you physically attacked? | |  | 0 time (0) 1 – 12 or more times (1) | |  | Physically attacked |
|  |  | | | **Social-level variables** | | |
| During the past 30 days, how often did your parents or guardians understand your problems and worries? | |  | Most of the time/always (1) Never/rarely/sometimes (0) | |  | Supportive parental figures |
| During the past 30 days, how often were most of the students in your school kind and helpful? | |  | Most of the time/always (1) Never/rarely/sometimes (0) | |  | Helpful peers |
| How many close friends do you have? | |  | 0 close friends (0) 1 close friends (1) 2 close friends (2) 3+ close friends (3) (coded continuous) | |  | Close friends |
| During the past 30 days, how often did you go hungry because there was not enough food in your home? | |  | Most of the time/always (1) Never/rarely/sometimes (0) | |  | Food insecurity |
