# Supplementary material for: Prevalence and correlates of physical fighting among adolescents in Paraguay: Findings from the 2017 national school-based health survey
Source: PLoS One. 2022 Dec 30;17(12):e0279402. doi: 10.1371/journal.pone.0279402 (PMC9803110; doi:10.1371/journal.pone.0279402)
Supplement: S2 Table — (DOCX) [file pone.0279402.s002.docx]

**Table 2: Cumulative proportion of factors in school-attending adolescents in Paraguay, GSHS 2017**

| **Variable** | **Cumulative Percentage [Unweighted Count]** | **Percent Not involved in physical fights [*Unweighted Count]** | **Percent Involved in physical fights [*Unweighted Count]** | **p-value** |
| --- | --- | --- | --- | --- |
| Age (SD) | 14.90 (1.58) [3,101] | 14.90 (1.59) [2,838] | 14.85 (1.43) [247] | 0.685 |
| Sex (Male) | 48.81 [3,087] | 46.94 [2,825] | 69.75 [247] | <0.001 |
| Anxiety | 9.47 [3,131] | 9.00 [2,866] | 15.15 [249] | 0.006 |
| Loneliness | 11.13 [3,090] | 10.52 [2,837] | 17.62 [241] | 0.004 |
| Food deprivation | 2.45 [3,113] | 2.45 [2,854] | 2.53 [245] | 0.929 |
| Close friends (SD) | 2.48 (0.89) [3,101] | 2.47 (0.89) [2,844] | 2.54 (0.89) [243] | 0.263 |
| Bullying victimization | 16.76 [2,974] | 15.41 [2,731] | 32.59 [231] | <0.001 |
| Truancy | 4.07 [3,109] | 3.59 [2,847] | 9.23 [248] | <0.001 |
| Physical Activity | 33.71 [3,066] | 32.12 [2,805] | 52.88 [247] | <0.001 |
| Sedentary | 34.02 [3,089] | 33.89 [2,837] | 35.85 [241] | 0.526 |
| Supportive parental figures | 49.15 [3,099] | 50.16 [2,842] | 37.58 [243] | 0.002 |
| Helpful peers | 62.81 [3,077] | 63.38 [2,824] | 56.03 [240] | 0.026 |
| Suicide planning | 13.28 [3,085] | 12.14 [2,830] | 25.42 [242] | <0.001 |
| Early sexual debut | 11.43 [3,041] | 10.20 [2,792] | 26.14 [233] | <0.001 |
| Alcohol use | 35.27 [3,040] | 33.11 [2,781] | 60.07 [244] | <0.001 |
| Attacked | 15.41 [3,101] | 12.82 [2,841] | 44.37 [248] | <0.001 |

Notes:

All variables are expressed as percentages with the exception of age and close friends, which are expressed as mean and standard deviation.

The square brackets show the unweighted counts, while all the reported percentages are weighted; the results of tests and their p-values are all based on the survey versions of Pearson chi-square tests, which accounts for the complex survey design, to examine the differences in % involvement in physical fight status across each variable.

The total number of respondents was 3,149. Differences across each variable are due to missing records in the variables.

*For involvement in physical fights 18 records were missing, as such the totals for each variable by physical fighting status are a little different from the totals reported in these two columns. This is because these two columns show the results where the variable, as well as the physical fighting status records, are available.
