# Supplementary material for: Prevalence and correlates of physical fighting among adolescents in Paraguay: Findings from the 2017 national school-based health survey
Source: PLoS One. 2022 Dec 30;17(12):e0279402. doi: 10.1371/journal.pone.0279402 (PMC9803110; doi:10.1371/journal.pone.0279402)
Supplement: S3 Table — (DOCX) [file pone.0279402.s003.docx]

**Table 3: Unadjusted odds ratios, adjusted odds ratios for age and sex, and adjusted odds ratios for all statistically significant variables for the association of physical fights with selected attributes among school-attending adolescents in Paraguay, GSHS 2017**

|  | **Simple Logistic Regression Analysis** | | | **Multivariable Logistic Regression Analysis - Adjusted for Age and Sex (Model 1*)** | | | **Multivariable Logistic Regression Analysis - Adjusted for all variables significant in simple logistic regression model (Model 2**)** | | |
| --- | --- | --- | --- | --- | --- | --- | --- | --- | --- |
| **Variables** | **Unadjusted OR** | **95 % CI** | **p-value** | **Adjusted OR** | **95% CI** | **p-value** | **Adjusted OR** | **95 % CI** | **p-value** |
| Age | 0.98 | 0.87-1.10 | 0.688 | 0.97 | 0.86-1.09 | 0.542 | N/A |  |  |
| Sex (Male) | 2.61 | 2.05-3.32 | <0.001 | 2.66 | 2.10-3.38 | <0.001 | 2.45 | 1.87-3.20 | <0.001 |
| Anxiety | 1.81 | 1.20-2.72 | 0.006 | 2.15 | 1.44-3.21 | 0.001 | 1.04 | 0.49-2.22 | 0.911 |
| Loneliness | 1.82 | 1.23-2.70 | 0.004 | 2.20 | 1.48-3.27 | <0.001 | 1.31 | 0.79-218 | 0.288 |
| Food deprivation | 1.03 | 0.49-2.20 | 0.929 | 0.99 | 0.47-2.11 | 0.994 | N/A |  |  |
| Close friends | 1.10 | 0.92-1.31 | 0.299 | 1.04 | 0.86-1.25 | 0.695 | N/A |  |  |
| Bullying victimization | 2.65 | 2.03-3.47 | <0.001 | 2.55 | 1.92-3.80 | <0.001 | 1.31 | 0.97-1.76 | 0.077 |
| Truancy | 2.73 | 1.90-3.94 | <0.001 | 2.64 | 1.70-4.11 | <0.001 | 1.90 | 0.99-3.61 | 0.051 |
| Physical Activity | 2.37 | 1.77-3.18 | <0001 | 2.04 | 1.51-2.75 | <0.001 | 2.16 | 1.45-3.22 | <0.001 |
| Sedentary | 1.09 | 0.83-1.44 | 0.527 | 1.19 | 0.90-1.58 | 0.219 | N/A |  |  |
| Supportive parental figures | 0.60 | 0.44-0.81 | 0.002 | 0.55 | 0.40-0.75 | 0.001 | 0.90 | 0.62-1.31 | 0.568 |
| Helpful peers | 0.74 | 0.56-0.96 | 0.026 | 0.71 | 0.56-0.91 | 0.008 | 0.93 | 0.69-1.25 | 0.607 |
| Suicide planning | 2.47 | 1.90-3.20 | <0.001 | 2.98 | 2.34-3.80 | <0.001 | 1.89 | 1.28-2.80 | 0.003 |
| Early sexual debut | 3.11 | 2.32-4.19 | <0.001 | 2.64 | 1.94-3.59 | <0.001 | 1.90 | 1.28-2.84 | 0.003 |
| Alcohol use | 3.04 | 2.40-3.84 | <0.001 | 3.32 | 2.68-4.10 | <0.001 | 1.95 | 1.48-2.57 | <0.001 |
| Attacked | 5.42 | 4.07-7.23 | <0.001 | 4.97 | 3.67-6.72 | <0.001 | 3.29 | 2.17-4.99 | <0.001 |

OR, Odds Ratio; 95% CI, 95% Confidence Interval.

Note: Table 3 shows the results of design-based logistic regression models which took into account the complex survey design.

* All estimates are adjusted for age and sex; age and sex are each adjusted for the other.

** All estimates are adjusted for all variables listed in the model 2.
