# Supplementary material for: Prevalence and correlates of physical fighting among adolescents in Paraguay: Findings from the 2017 national school-based health survey
Source: PLoS One. 2022 Dec 30;17(12):e0279402. doi: 10.1371/journal.pone.0279402 (PMC9803110; doi:10.1371/journal.pone.0279402)
Supplement: Attachment — Submitted filename: Manuscript PONE-D-21-03008 Rev 09-11-2021.pdf [file pone.0279402.s004.pdf]

Manuscript: Physical fighting and associated factors among adolescents in Paraguay the 2017 national school-based health survey

Specific comments are:

#1 Page-4, Ln 75: *'Accelerated Action for the Health of Adolescents (AA-HA!)* –there is a typo in bracket – delete the sign (!).

# 2 Page-5, Ln 84-87: *"A recent study… over one third of adolescents …, it comes to boys, the prevalence increased to 41% in physically attacked and 46% in physical fighting".* It looks rates for boys are substantially higher than overall rates of one third – in this instance I recommend to specify rates for both girls and boys respectively– that will be helpful for readers and policy makers to take initiatives to reducing the gender gap.

#3 Page-5, Ln 100: "… causes of deaths and *DALYs* …". DALYs need to be spelled out at least once.

#4 Page-6, Ln 104: "... following Lao *PDR*, Cambodia …". PDR need to be spelled out.

#5 Page-8, Table 1: "How many close friends do you have? 0 close friends (0) 1 close friends (1) 2 close friends (2) 3+ close friends (3) *(coded continuous)"* – since there are only 3 values – author can say it is categorical.

#6 Page-9, Ln 155-156: *"Similar to previous studies, multivariable logistic regression models were developed to identify factors associated with physical fighting [10-14].* Instead of referring readers to look at previous studies [10-14] – the author can simply say due to dichotomous nature of the outcome variable 'physical fighting (no=0, yes=1), multivariable binary logistic regression models were developed to identify factors associated with physical fighting [give reference of Stat Book; e.g. Applied Logistic Regression by David W. Hosmer, Rodney X. Sturdivant and Stanley Lemeshow]

#7 Page-9, Ln 159-160: *"Odds ratios and their statistical significance, using the significance level of less than five Percent…".* The author can include ($p<0.05$) i.e. 'five Percent' should be 'five percent ($p<0.05$)'.

#8 Page 9-11, Results: The authors used unweighted sample for basic prevalence: 8.0% (unweighted count: 251)….; among males and females the proportion of physical fighting involvement was 11.4%, and 4.7%, respectively. Whereas overall sample characteristics in Table 2 is based on weighted data: Column 2 and overall Table 2 is confusing – does not match with actual prevalence for subgroups. In Statistical analysis section - authors did not mention anything about data weighting – how the weights were calculated. As the methods section did not mention about weighting – it assumed that the Multivariable logistic regression analysis was done based on unweighted data; and so did the unadjusted Odds Ratios (ORs). If the ORs are based on unweighted data – I strongly recommend to presented unweighted data in Table 2. Otherwise authors need to mention in methods section how data weighting used for both descriptive and logistic regression analysis.

#9 Page 12, Ln 193-94: *".. using simple binary logistic regression models".* This should be "… simple univariate binary logistic regression models".

#10 Page 12, Ln 194-95*: "The results of bivariate analyses show that out of sixteen attributes, four were not statistically significantly associated …".* The author did not show the bivariate analysis for prevalence of physical fights with significant labels. I recommend authors to reformat the Table 2 in following format based on unweighted data - including both sample characteristics and prevalence of physical fights for each variable category with p-values (bivariate analysis).

Suggested format for Table 2: Sample characteristics and percentage involved in any physical fights with selected attributes among school-attending adolescents in Paraguay, GSHS 2017

Variable	Total respondents: % (n)	Involved in any physical fights: % (n)
All	100.0 (3131)	8.0 (251)
Age		XX.X
<17 years		
17-18 years		
Mean (Sd)		-
p-values		*p-values*
Gender		
Female	51.2 (XX)	4.7 (xx)
Male	48.8 (XX)	11.4 (xx)
		p-values
Anxiety		
No		
Yes		
p-values		*p-values*
Number of close friends		
None		
One		
Two		
Three or more		*p-values*
p-values		
Last 12 months how many times physically attacked		
None		
One or more times		
p-values		*p-values*

Note p-values are calculated to examine the differences for % involved in physical fight across groups
Other than age no need for SD

#11 Page 13, Title "Table 3: *Unadjusted odds ratios for the association …Paraguay, GSHS 2017".*
The author can reward the title as "Table 3: … odds ratios from univariate binary logistic regression models for the association … Paraguay, GSHS 2017".

#12 Page 13, Table 3: Reference category for none of the variables are indicated. Reference category for each variable should be included.

#13 Page 13, Table 3: In variable column - SD included with Age [Age(SD)] and Close friends [Close friends (SD)]; male added with Sex [Sex (male)] – this typos need to be corrected.

#14 Page 13, Table 3: In footnote indicated *"All estimates are adjusted for age and sex; age; or sex".*
This is confusing because these ORs are unadjusted from univariate binary logistic regression analysis.

#15 Page 15, Title *"Table 4: Multivariate analysis…. adolescents in Paraguay, GSHS 2017".*
There are many types of multivariate analysis. It's need be specific that "Multivariable logistic regression analysis…. adolescents in Paraguay, GSHS 2017"

#16 Page 15, Table 4: Reference category for none of the variables are indicated. Reference category for each variable should be included.

#17 Page 15, Table 4: In variable column - SD included with Age [Age(SD)] and Close friends [Close friends (SD)]; male added with Sex [Sex (male)] – this typos need to be corrected.

#18 Page 16, Table 4: In footnote indicated *"All estimates are adjusted for age and sex; age; or sex"*. This is confusing because this kind of adjusted analysis for "age and sex"; "age; or sex" -requires series of multivariable logistic regression models. I believe authors did just one Multivariable logistic regression analysis. So the footnote should be "All estimates are adjusted for all variables included in the Multivariable logistic regression model".

#19 Page 17, Title *"Table 5: Outcomes of multivariable analysis of variables ...., GSHS 2017"*. There are many types of multivariable analysis. So the authors need be specific that "Multivariable logistic regression analysis…., GSHS 2017".

#20 Page 17, Table 5: Reference category for none of the variables are indicated. Reference category for each variable should be included.

#21 Page 13, 15, 17: Tables 3 to 5: Instead of using three Tables on ORs – the authors can presents results of Tables 3, 4 and 5 as one Table – so the readers can see the differences of unadjusted and adjusted ORs side by side in same Table with following format below. The new Table can be titled as "Table 3: Unadjusted odds ratios (ORs) from univariate logistic regression analysis and adjusted ORs from multivariable logistic regression analysis with 95% confidence interval (95%CI) for the association … Paraguay, GSHS 2017

Variables	Univariate logistic regression analysis		Multivariable logistic regression analysis: Model 1		Multivariable logistic regression analysis: Model 2	
	Unadjusted OR (95CI)	p-values	Adjusted OR (95CI)	p-values	Adjusted OR (95CI)	p-values
Age (cont. 13-18 years)	0.97 (0.86-1.09)	0.542	0.97 (0.86-1.09)	0.542	NS in Model 1	
Sex (ref. cat: female)	2.66 (2.10-3.38)	<0.001	2.66 (2.10-3.38)	<0.001	2.45 (1.87-3.20)	<0.001
Food deprivation		0.994		0.994	NS in Model 1	
Anxiety (ref. cat: no)						
No. of close friends (ref. category: none)		0.695		0.695	NS in Model 1	
Physically attacked (ref. cat: none)	2.04 (1.51-2.75)	0.219	4.97 (3.67-6.72)	<0.001	3.29 (2.17-4.99)	<0.001

Model 1: All predictor variables included in the multivariable logistic regression Model 1;
Model 2: Variables found significant (p<0.05) in Model 1 are included in the multivariable logistic regression model.
Adjusted ORs in Model 1 and Model 2: All estimates are adjusted for all variables included in the multivariable logistic regression model.
