# Supplementary material for: Prevalence and correlates of physical fighting among adolescents in Paraguay: Findings from the 2017 national school-based health survey
Source: PLoS One. 2022 Dec 30;17(12):e0279402. doi: 10.1371/journal.pone.0279402 (PMC9803110; doi:10.1371/journal.pone.0279402)
Supplement: Attachment — Submitted filename: PONE-D-21-03008 Review.docx [file pone.0279402.s005.docx]

**Title of the manuscript:** Physical fighting and associated factors among adolescents in Paraguay the 2017 national school-based health survey

Thank you for the opportunity to review this paper. The aim of this study was to investigate the prevalence and correlates of physical fighting among adolescents in Paraguay based on the 2017 national school-based health survey. The manuscript addresses an interesting topic; however, there are some issues that need to be improved prior to publication. I believe this manuscript can be considered for publication after addressing the comments below:

**Title:** Physical fighting and associated factors among adolescents in Paraguay the 2017 national school-based health survey.

I suggest modifying the title to *“Prevalence and correlates of Physical fighting among adolescents in Paraguay: Findings from the 2017 national school-based health survey.”*

**Abstract:**

1. The English grammar and style should be checked throughout the manuscript.

Background: Line 28 – Causes of deaths – spelling error. Please change to causes of death.

2. Lines 30-31 – Sentence seems unfinished. The extent to which previously identified factors ..what?

3. Lines 43-44 – Please combine as one sentence.

**Introduction:**

1. The English grammar and style should be checked throughout the manuscript.

2. Lines 83-84 “tells us that” – too colloquial? Perhaps change to something formal.

3. Lines 87 – “41% in physically attacked and 46% in physical fighting” – word missing - change to *“41% in being physically attacked and 46% involved in physical fighting”*

4. Line 102 – change to *“at least one physical fight in the last 12 months”*

**Methods**:

1. No mention of study design used. I strongly recommend the authors to refer to STROBE checklist for cross-sectional/observational studies <https://www.strobe-statement.org/>

2. Key information such as number of schools, age of the participants included in the analysis and the eligibility criteria are missing in this section.

Lines 122-123 – Combine both sentences.

3. Lines 135-138 – What is the rationale behind classifying two or more fights as having participated in a physical fight and not one or more fights?

The sentence “two or more fights as having participated in **a** physical fight” in itself is contradictory.

4. Line 163 – Incomplete sentence. Please check grammar and sentencing throughout.

5. Statistical analysis must talk about descriptive analysis of baseline characteristics (presented in Table 2) followed by the main analysis.

6. How was multicollinearity checked? Please elaborate with the type of analysis.

**Results:**

1. Table 2 – Indicate number of participants along with percentages for each category and the number with missing data for each variable of interest. Please refer to the STROBE checklist.

2. I suggest conducting Pearson’s chi-square test to check if the characteristics of those involved in physical fights were different from those who were not.

3. Lines 206 – 12 out of 16 variables were significantly associate – not 14.

4. Line 217 – Typo – Uppercase “T” for table 3.

5. Table 5 – Sex to be modified to Sex (Male).

6. Tables 2-5 – I would suggest showing both categories for each variable for clarity and readability. For example, in Table 5,

Currently, it is not a table with standalone information, we need refer back to Table 1.

| **Variable** | **Adjusted OR** | **95% CI** | **p-value** |
| --- | --- | --- | --- |
| **Anxiety** |  |  |  |
| Never/rarely/sometimes | 1.00 | Reference category |  |
| Most of the time/always | 1.04 | 0.49-2.22 | 0.911 |

**Discussion:**

1. Lines 252-253 – Rephrase the sentence to “Consistent with other studies,…”

2. Lines 274 – I would suggest using the word “association” or “odds” given a regression analysis was conducted rather than use of term “correlation”

3. Lines 332-335 – Please expound on what the other sorts of interpersonal violence are based on your literature review.

4. Lines 351- Please change to …accelerate actions for “them”

5. The findings have been critically appraised with other literature/research findings, however, the “so what” factor, that is, implications of study findings to individuals/community and policy is vague.

**Conclusion:**

1. Lines 354-355 – Useful how? Please rephrase to say how the findings may be useful to inform policy and planning. What intervention programs are in place as best practice in Paraguay or other countries/ what is recommended in other countries?
