# Supplementary material for: Prevalence and correlates of physical fighting among adolescents in Paraguay: Findings from the 2017 national school-based health survey
Source: PLoS One. 2022 Dec 30;17(12):e0279402. doi: 10.1371/journal.pone.0279402 (PMC9803110; doi:10.1371/journal.pone.0279402)
Supplement: Attachment — Submitted filename: Response to Reviewers 20220305.docx [file pone.0279402.s006.docx]

Manuscript ID: PONE-D-21-03008

Response to Reviewers

To

Dr Valsamma Eapen

Academic Editor

PLOS ONE

Dear Dr. Valsamma Eapen:

Thank you very much for your consideration of our manuscript, Physical fighting and associated factors among adolescents in Paraguay the 2017 national school-based health survey. We have revised the manuscript thoroughly according to the comments by the editor and reviewers. We addressed all points clearly in the revised manuscript.

Our responses to the editor and reviewers are provided in a separate sheet. Where we have changed the texts and the corresponding sentences in the texts have been highlighted using track changes.

- The yellow-highlight: the changed parts responding to the reviewers’ comments.

- The grey-highlight: the sentences or clauses which include the changes based on the English-language editor’s corrections and/or suggestions. We used the professional English-language service for the revised manuscript.

All line numbers referred to are in the margins of our revised manuscript with tracked changes.

We hope that the revisions are satisfactory in addressing issues raised by the editor and reviewers and look forward to hearing your decision about this article.

Yours sincerely,

Authors

**=============================================================**

**Journal Requirements:**

When submitting your revision, we need you to address these additional requirements.

1. Please ensure that your manuscript meets PLOS ONE's style requirements, including those for file naming. The PLOS ONE style templates can be found at https://journals.plos.org/plosone/s/file?id=wjVg/PLOSOne_formatting_sample_main_body.pdf and https://journals.plos.org/plosone/s/file?id=ba62/PLOSOne_formatting_sample_title_authors_affiliations.pdf.

We checked the PLOS ONE's style requirements and accordingly revised the manuscript.

2. Please refer to the specific statistical analyses performed as well as any post-hoc corrections to correct for multiple comparisons.

Responding to the reviewers’ comments, we mentioned the models and tests we used in the manuscript.

3. We suggest you thoroughly copyedit your manuscript for language usage, spelling, and grammar.

The name of the colleague or the details of the professional service that edited your manuscript

We, Hiroko Taniguchi and Masood Ali Shaikh, asked a professional English-language editor, Elizabeth Nyman, to edit the revised manuscript. We carefully checked every correction and/or suggestion and made further revisions to the manuscript.

Additional Editor Comments (if provided):

While the article is of interest, I agree with the reviewers that before it can be accepted for publication, it would require substantial revision particularly in terms of English and grammar corrections as well as alternate statistical analysis such as conducting Pearson’s chi-square test to check if the characteristics of those involved in physical fights were different from those who were not."

We have revised the manuscript thoroughly according to the comments by you and the reviewers. We addressed all points in the reponse below and the revised manuscript.

**=============================================================**

**Reviewer #1:** Please see the attached pdf file for Table format

Manuscript: Physical fighting and associated factors among adolescents in Paraguay the 2017 national school-based health survey

**Specific comments are:**

**#1 Page-4, Ln 75: ‘Accelerated Action for the Health of Adolescents (AA-HA!) –there is a typo in bracket – delete the sign (!).**

This is the official name of the guidance. Please find the WHO website and the cover of the publication for your information. <https://www.who.int/publications/i/item/9789241512343>

**# 2 Page-5, Ln 84-87: “A recent study… over one third of adolescents …, it comes to boys, the prevalence increased to 41% in physically attacked and 46% in physical fighting”. It looks rates for boys are substantially higher than overall rates of one third – in this instance I recommend to specify rates for both girls and boys respectively– that will be helpful for readers and policy makers to take initiatives to reducing the gender gap.**

Thank you for your advice. We specified the rates for boys and girls. (Page 5, line 189-190)

**#3 Page-5, Ln 100: “… causes of deaths and DALYs …”. DALYs need to be spelled out at least once.**

We spelled it out. (Page 6, line 293)

**#4 Page-6, Ln 104: “... following Lao PDR, Cambodia …”. PDR need to be spelled out.**

We spelled it out. (Page 6, line 298)

**#5 Page-8, Table 1: “How many close friends do you have? 0 close friends (0) 1 close friends (1) 2 close friends (2) 3+ close friends (3) (coded continuous)” – since there are only 3 values – author can say it is categorical.**

The reason for calling this variable “coded continuous” is that in the logistic regression model we used it as a continuous variable. Therefore, in table 3 we provided only one Odds Ratio (OR) for this covariate owing to the fact that it was used as a continuous variable as opposed to categorial; in which case one would use one category as a reference and provide ORs for all others. Also in table 2, we have provided mean and SD for this variable.

**#6 Page-9, Ln 155-156: “Similar to previous studies, multivariable logistic regression models were developed to identify factors associated with physical fighting [10-14]. Instead of referring readers to look at five previous studies [10-14] – the author can simply say due to dichotomous nature of the outcome variable ‘physical fighting (no=0, yes=1), multivariable binary logistic regression models were developed to identify factors associated with physical fighting [give reference of Stat Book; e.g. Applied Logistic Regression by David W. Hosmer, Rodney X. Sturdivant and Stanley Lemeshow]**

Thanks. In the revised manuscript we have incorporated the comment as under: (Page 9, line 581-583)

Due to the dichotomous nature of the outcome variable ‘physical fighting (no=0, yes=1), multivariable binary logistic regression models were developed to identify the factors associated with physical fighting [*REFERENCE*: David W. Hosmer Jr., Stanley Lemeshow, Rodney X. Sturdivant. Applied Logistic Regression. 3rd ed., John Wiley & Sons, 2013].

**#7 Page-9, Ln 159-160: “Odds ratios and their statistical significance, using the significance level of less than five Percent…”. The author can include (p<0.05) i.e. ‘five Percent’ should be ‘five percent (p<0.05)’.**

Thanks. In the revised manuscript we have incorporated the comment as under: (Page 9, line 586)

‘Odds ratios and their statistical significance, using the significance level of less than five percent **(p<0.05)**, for the association between physical fight involvement with the independent variables were analyzed using survey version of binary simple logistic regression models.’

**#8 Page 9-11, Results: The authors used unweighted sample for basic prevalence: 8.0% (unweighted count: 251)….; among males and females the proportion of physical fighting involvement was 11.4%, and 4.7%, respectively. Whereas overall sample characteristics in Table 2 is based on weighted data: Column 2 and overall Table 2 is confusing – does not match with actual prevalence for subgroups.** **In Statistical analysis section - authors did not mention anything about data weighting – how the weights were calculated. As the methods section did not mention about weighting – it is assumed that the Multivariable logistic regression analysis was done based on unweighted data; and so did the unadjusted Odds Ratios (ORs). If the ORs are based on unweighted data – I strongly recommend to presented unweighted data in Table 2. Otherwise authors need to mention in methods section how data weighting constructed for both descriptive and logistic regression analysis; in that case prevalence should be based on weighted data.**

Kindly note that on:

Page 9, line 587-588, we stated that “… were analysed using a survey version of binary simple logistic regression models.”

Page 9, line 590-591, we stated that “This step was followed by two additional survey binary multivariable logistic regression models.”

Page 10, line 633-634, we stated that “All proportions – expressed in percentages – are weighted”.

The lines from the original manuscript submitted specify that all analyses involved survey versions of the logistic regression models and that all percentages reported are weighted. So, unless otherwise specified, we used design-based analyses. Hence all analyses incorporated – took into account – survey design of the GSHS.

Hence, we respectfully, do not concur with some of the comments specified here, and our responses to each statement are as under please:

**(a) “The authors used unweighted sample for basic prevalence: 8.0% (unweighted count: 251)….; among males and females the proportion of physical fighting involvement was 11.4%, and 4.7%, respectively.”**

We used a weighted sample and provided weighted prevalence i.e. 8.0%, 11.4%, 4.7%. However, we also provided an unweighted count of 251; which is the number of students who were involved in two or more fights.

**(b) “Column 2 and overall Table 2 is confusing – does not match with actual prevalence for subgroups.”**

In this table, Column 2 provides cumulative i.e. overall percentages; so overall in the survey 48.81% of respondents were males. Column 3 i.e. ‘percent not involved in physical fights’ refers to the subgroup that was not involved in physical fights and in this subgroup males comprised 46.94%, while the rest were females. Column 4 i.e. ‘Percent involved in physical fights’ refers to the subgroup that was involved in physical fights, and in this subgroup, males comprised 69.75%.

**(c) “In Statistical analysis section - authors did not mention anything about data weighting – how the weights were calculated.”**

Since this was a secondary analysis of the GSHS data. We used the survey design variables i.e. weights, strata and PSU, that were provided with the dataset and were calculated by the agencies that conducted this survey. Their detailed methodology is available on the website from where we downloaded this dataset. We provided the weblink in addition to stating that details on the methodology of the survey are also provided there. (Page 7, line 417)

**(d) As the methods section did not mention about weighting – it is assumed that the Multivariable logistic regression analysis was done based on unweighted data; and so did the unadjusted Odds Ratios (ORs). If the ORs are based on unweighted data – I strongly recommend to presented unweighted data in Table 2. Otherwise authors need to mention in methods section how data weighting constructed for both descriptive and logistic regression analysis; in that case prevalence should be based on weighted data.**

As stated above, we mentioned in the methods section that analyses are weighted. The “unadjusted Odds Ratios” refers to the use of simple logistic regression models as part of bivariate analysis i.e. the simple logistic regression models only had one explanatory/independent variable. As such the resulting odds ratios were unadjusted for any other explanatory variables. The multivariable models had more than one explanatory variable, and as such, each explanatory variable was adjusted for all the other explanatory variables in the models.

**#9 Page 12, Ln 193-94: “.. using simple binary logistic regression models”. This should be “… simple univariate binary logistic regression models”.**

On Page 12, lines 687-689, We stated that “Column 2 of Table 3 shows the odds ratios of the individual association of involvement in physical fights with all sixteen selected factors, and their statistical significance using simple binary logistic regression models. **The results of the bivariate analyses show that** …”

If we revise the statement as per comment the second sentence (highlighted in bold above) would follow by talking about ‘bivariate analysis.’ We request that “simple binary logistic regression’ be kept as such, as the term “simple” logistic regression model implies that only one explanatory variable is in the model.

**#10 Page 12, Ln 194-95: “The results of bivariate analyses show that out of sixteen attributes, four were not statistically significantly associated …”. The author did not show the bivariate analysis for prevalence of physical fights with significant labels. I recommend authors to reformat the Table 2 in following format based on unweighted data - including both sample characteristics and prevalence of physical fights for each variable category with p-values (bivariate analysis).**

**Suggested format for Table 2: Sample characteristics and percentage involved in any physical fights with selected attributes among school-attending adolescents in Paraguay, GSHS 2017 [see the attached pdf file]**

Table 2 has been revised as per the comment in the revised manuscript. (Page 11)

**#11 Page 13, Title “Table 3: Unadjusted odds ratios for the association …Paraguay, GSHS 2017”.**

**The author can reward the title as “Table 3: … odds ratios from univariate binary logistic regression models for the association … Paraguay, GSHS 2017”.**

The “univariate” implies analysis pertaining to one variable only. However, a simple logistic regression model with two variables i.e. one dependent and the other independent is a bivariate analysis. We used the term ‘unadjusted’ to clearly state that the analysis reported in this pertains of each explanatory variable’s association with the outcome variable. As such all coefficients are “unadjusted”. (Page 13)

**#12 Page 13, Table 3: Reference category for none of the variables are indicated. Reference category for each variable should be included.**

All explanatory variables used are binary (1/0 coded) variables. It is respectfully pointed out that when reporting binary variables, it is the statistical norm that presence/yes/1 is compared against absence/no/0.

**#13 Page 13, Table 3: In variable column - SD included with Age [Age(SD)] and Close friends [Close friends (SD)]; male added with Sex [Sex (male)] – this typos need to be corrected.**

Thank you for pointing this out. In Table 3 in the revised manuscript these types have been corrected and deleted. However, “sex (male)” implies that males are compared against females, so we suggest keeping it as it is, please. (Page 13)

**#14 Page 13, Table 3: In footnote indicated “All estimates are adjusted for age and sex; age; or sex”. This is confusing because these ORs are unadjusted from univariate binary logistic regression analysis.**

Thanks indeed for pointing this out. This error was the result of copying/pasting from the original table 4, which was a mistake. In the revised manuscript this footnote is kept only for Column 3 (the original table 4). (Page 13, revised Table 3, line 705)

**#15 Page 15, Title “Table 4: Multivariate analysis…. adolescents in Paraguay, GSHS 2017”.**

**There are many types of multivariate analysis. It’s need be specific that “Multivariable logistic regression analysis…. adolescents in Paraguay, GSHS 2017”**

Thanks for pointing this out. In the revised manuscript, the “Multivariate” has been replaced with ‘Multivariable’. (Page 13, revised Table 3, Column 3)

**#16 Page 15, Table 4: Reference category for none of the variables are indicated. Reference category for each variable should be included.**

All explanatory variables used are binary (1/0 coded) variables. It is respectfully pointed out that when reporting binary variables, it is the statistical norm that presence/yes/1 is compared against absence/no/0. (Page 13, revised Table 3, Column 3)

\#**17 Page 15, Table 4: In variable column - SD included with Age [Age(SD)] and Close friends [Close friends (SD)]; male added with Sex [Sex (male)] – this typos need to be corrected.**

Thank you for pointing this out. In the revised manuscript these types have been corrected and deleted. However, “sex (male)” implies that males are compared against females, so we suggest keeping it as it is, please. (Page 13, revised Table 3)

**#18 Page 16, Table 4: In footnote indicated “All estimates are adjusted for age and sex; age; or sex”.**

**This is confusing because this kind of adjusted analysis for “age and sex”; “age; or sex” -requires series of multivariable logistic regression models. I believe authors did just one Multivariable logistic regression analysis. So the footnote should be “All estimates are adjusted for all variables included in the Multivariable logistic regression model”.**

We did in fact run a series of multivariable logistic regression models. This was pointed out as below.

The first set of models adjusted only for age and sex. (Page 9, line 592-593)

We also rewrote the footnote of Column 3 of Table 3 (originally Table 4) and relevant main text as below in order to make this point clearer:

*All estimates are adjusted for age and sex; age and sex are each adjusted for the other. (Page 13. line 705)

Column 3 of Table 3 shows the analysis of all the attributes studied for age and sex . The sex and age variables were each, adjusted for the other. (Page 14, line 708-709)

**#19 Page 17, Title “Table 5: Outcomes of multivariable analysis of variables …., GSHS 2017”.**

**There are many types of multivariable analysis. So the authors need be specific that “Multivariable logistic regression analysis…., GSHS 2017”.**

Thanks indeed for pointing this out. In the revised manuscript the title has been revised accordingly. And the table 3, 4, and 5 have been merged as per another comment. (Page 13, revised Table 3)

**#20 Page 17, Table 5: Reference category for none of the variables are indicated. Reference category for each variable should be included.**

All explanatory variables used are binary (1/0 coded) variables. It is respectfully pointed out that when reporting binary variables, it is the statistical norm that presence/yes/1 is compared against absence/no/0.

**#21 Page 13, 15, 17: Tables 3 to 5: Instead of using three Tables on ORs – the authors can presents results of Tables 3, 4 and 5 as one Table – so the readers can see the differences of unadjusted and adjusted ORs side by side in same Table with following format below. The new Table can be titled as**

**“Table 3: Unadjusted odds ratios (ORs) from univariate logistic regression analysis and adjusted ORs from multivariable logistic regression analysis with 95% confidence interval (95%CI) for the association … Paraguay, GSHS 2017 [[see the attached pdf file]**

Tables 3-5 have been combined as per comment in the revised manuscript. (Page 13, revised Table 3)

**=============================================================**

**Reviewer #2:** **Title of the manuscript: Physical fighting and associated factors among adolescents in Paraguay the 2017 national school-based health survey**

**Thank you for the opportunity to review this paper. The aim of this study was to investigate the prevalence and correlates of physical fighting among adolescents in Paraguay based on the 2017 national school-based health survey. The manuscript addresses an interesting topic; however, there are some issues that need to be improved prior to publication. I believe this manuscript can be considered for publication after addressing the comments below:**

**Title: Physical fighting and associated factors among adolescents in Paraguay the 2017 national school-based health survey.**

**I suggest modifying the title to “****Prevalence and correlates of Physical fighting among adolescents in Paraguay: Findings from the 2017 national school-based health survey.”**

We have revised the title accordingly. Thank you. (Page 1, line 2-3)

**Abstract:**

**1. The English grammar and style should be checked throughout the manuscript.**

We asked a professional English-language editor to edit the revised manuscript. We carefully checked every correction and/or suggestion and made further revisions to the manuscript.

Background: Line 28 – Causes of deaths – spelling error. Please change to causes of death.

We changed it to causes of death. (Page 2, line 31)

**2.** Lines 30-31 – Sentence seems unfinished. The extent to which previously identified factors ..what?

We rewrote it as “the extent to which previously identified factors correlate physical fighting.” (Page 2, line 34)

**3.** Lines 43-44 – Please combine as one sentence.

We combined the first and second sentences into one sentence. (Page 2, line 46-47)

**Introduction:**

**1. The English grammar and style should be checked throughout the manuscript.**

We asked a professional English-language editor to edit the revised manuscript. We carefully checked every correction and/or suggestion and made further revisions to the manuscript.

2. Lines 83-84 “tells us that” – too colloquial? Perhaps change to something formal.

We changed it to “shows.” (Page 5, line 185)

**3. Lines 87 – “41% in physically attacked and 46% in physical fighting” – word missing - change to “41% in being physically attacked and 46% involved in physical fighting”**

Based on another reviewer’s advice, we rewrote this part as below. (Page 5, line 189-190)

the prevalence was higher among boys than girls for physically attacked (41%, 29%) and physical fighting (46%, 27%) respectively

**4. Line 102 – change to “at least one physical fight in the last 12 months”**

We changed it to “at least one physical fight in the last 12 month.” (Page 6, line 296)

**Materials and Methods:**

**1. No mention of study design used. I strongly recommend the authors to refer to STROBE checklist for cross-sectional/observational studies https://www.strobe-statement.org/**

The comment has been addressed in the revised manuscript as below. (Page 6, lines 309-310)

This methodology involves cross-sectional surveys using a self-administered questionnaire.

**2. Key information such as number of schools, age of the participants included in the analysis and the eligibility criteria are missing in this section.**

Thanks for pointing this out!

**Age:** The age and its coding has been revised to more clearly reflect how ages were originally provided with the dataset and how we coded it for our analysis.

There were 23 records for which age was provided in the dataset as ‘less than 11 years’ and for 122 records for which age was provided as 18 years or older. This has been clarified in Table 1, in the revised manuscript. (Page 8, Table 1)

**Eligibility criteria:** Kindly find a new, following paragraph (Page 6, line 311-315)

GSHSs use a two-stage cluster sample design to produce representative data for all students in the specified grades in the specified year groups. The first stage entails the selection of the schools with a probability proportional to the enrolment size, and the second stage classes are randomly selected. All students present in the selected classes on the day of the survey administration are eligible to participate in the survey.

**Number of schools:** Each country that conducts GSHS is supposed to produce a country report detailing among other things, the total number of schools selected. However, a re-check on February 15 2022, does not show this report from Paraguay for the 2017 GSHS. However, schools are selected to represent the entire country, and GSHSs being two-stage cluster surveys, select schools in the first stage with probability proportional to enrollment size.

**Lines 122-123 – Combine both sentences.**

The two sentences are combined in the revised manuscript as under: (Page 7, line 419-420)

Paraguay, 3,149 students at Octavo-Tercer Curso completed the survey questionnaire; the school, student, and total response rates as 100%, 87%, and 87%, respectively.

**3. Lines 135-138 – What is the rationale behind classifying two or more fights as having participated in a physical fight and not one or more fights?**

**The sentence “two or more fights as having participated in a physical fight” in itself is contradictory.**

It boils down to attributes of adolescent behaviour and adaptive socialization. In many ways, they are still learning how to appropriately deal with disagreement, empathy and disappointment. Thus, the occasional push or shove should not be considered problematic behaviour within the context of correlates for serious behavioural and/or mental pathology. Also, in particular for boys, within this adaptive process, some aggressivity is in many ways part of their development until they learn appropriate ways of dealing with such things as a disappointment.

What we really want to see is the truly problematic behaviour, such as involvement in physical fights repeatedly within a period of recall.

Several published studies using GSHSs have also used involvement in 2 or more physical fights as constituting such involvement.

**4. Line 163 – Incomplete sentence. Please check grammar and sentencing throughout.**

This sentence has been updated in the revised manuscript as below. (Page 9, line 597-598)

This step was followed by two additional survey binary multivariable logistic regression models.

**5. Statistical analysis must talk about descriptive analysis of baseline characteristics (presented in Table 2) followed by the main analysis.**

In ‘Statistical analysis’ in ‘Methods’ section it was stated as below: (Page 9, line 590-592)

“First, the distribution of selected independent variables in the entire sample was examined, followed by the distribution of selected independent variables within the dichotomised physical fights involvement variable.”

We will be grateful for more elaboration as to what additional information needs to be added here, please.

**6. How was multicollinearity checked? Please elaborate with the type of analysis.**

We used the survey version of Variance Inflation Factor (VIF). The VIF was considered acceptable if it was less than 10. In our final regression model (model 2 – as provided in the revised table 3) VIF was less than 3 for each explanatory variable.

**Results:**

**1. Table 2 – Indicate number of participants along with percentages for each category and the number with missing data for each variable of interest. Please refer to the STROBE checklist.**

Table 2 has been revised accordingly. (Page 11)

**2. I suggest conducting Pearson’s chi-square test to check if the characteristics of those involved in physical fights were different from those who were not.**

Table 2 has been revised accordingly. (Page 11)

**3. Lines 206 – 12 out of 16 variables were significantly associate – not 14.**

Thanks for pointing this out! In the revised manuscript, this mistake has been corrected. (Page14, line 710)

**4. Line 217 – Typo – Uppercase “T” for table 3.**

The tables 3 to 5 have been merged together as per the comment of the other reviewer as well, and this typo has been corrected. (Page 14, line 708)

**5. Table 5 – Sex to be modified to Sex (Male).**

This typo has been corrected in the revised manuscript. (Page 13)

**6. Tables 2-5 – I would suggest showing both categories for each variable for clarity and readability. For example, in Table 5,**

**Currently, it is not a table with standalone information, we need refer back to Table 1.**

| **Variable** | **Adjusted OR** | **95% CI** | **p-value** |
| --- | --- | --- | --- |
| **Anxiety** |  |  |  |
| Never/rarely/sometimes | 1.00 | Reference category |  |
| Most of the time/always | 1.04 | 0.49-2.22 | 0.911 |

The tables 3 to 5 have been merged together as per the comment of the other reviewer as well, and this typo has been corrected. (Page 13)

Since there are only three tables and in order to avoid presenting a rather crowded table, it would be easier for the reader to refer to just one table when reading the other two tables.

**Discussion:**

**1.** Lines 252-253 – Rephrase the sentence to “Consistent with other studies,…”

We changed “In common with many studies with various methods” to “Consistent with other studies.” (Page 15, line 793)

2. Lines 274 – I would suggest using the word “association” or “odds” given a regression analysis was conducted rather than use of term “correlation”

We changed this to association. (Page 16, line 850)

We also changed the other two “correlation” to association(s) as below:

Moreover, other studies identified **the associations** between alcohol use, early sexual debut, and suicide planning (41, 42). (Page 16, line 858)

This study found that having been physically attacked shows the highest association with physical fighting among the variables, while **the association** between being bullied and physical fighting was not statistically significant in the analysis adjusted for all variables. (Page 18, line 960)

3. Lines 332-335 – Please expound on what the other sorts of interpersonal violence are based on your literature review.

We added the other sorts of interpersonal violence in this sentence which are mentioned in the Introduction section. It includes bullying, emotional abuse, sexual and physical assault, and homicide. (Page 19, line 992-994)

**4. Lines 351- Please change to …****accelerate actions for “them”**

Based on the English-language editor’s suggestion, we rewrote this part as below: (Page 19, line 1012-1013)

We hope the study could be useful for policy makers when developing health strategies and plans for adolescents and in accelerating such actions.

**5. The findings have been critically appraised with other literature/research findings, however, the “so what” factor, that is, implications of study findings to individuals/community and policy is vague.**

Thank you very much for your comments. In the Discussion session, our study highlights a couple of implications as below. To make these implications clearer, we added the summary of those points into the Conclusion section. (Page 20, line 1051-1061)

***

1. Policy and community levels: strengthening social norms, values, and practical response

To make progress in the prevention of violence and associated factors such as alcohol use, early sexual debut, and suicide planning, Paraguay has shown better progress in the areas of implementation and enforcement of laws, caregiver support, and school-based preventions than other upper middle-income countries in the same regions. However, Paraguay lags behind in strengthening social norms and values as well as practical responses such as mental health services for both victims and perpetrators. Those areas should be strengthened. (Kindly refer to the highlighted part on Page 17, line 896-907)

2. Policy, community and family/individual levels: promoting parental support

Although the Law to prohibit all corporal punishment of children was approved and enacted in Paraguay in 2016, many families still regard parents’ corporal punishment as a teaching measure. Considering the facts that the countries with bans on corporal punishment in schools and at home have seen less physical fighting and that parental support is seen as a preventive attribute in the studies, Paraguay should also move ahead in making shifts in the relationship between parents and children by tireless awareness raising and practical executions of legislation. (Kindly refer to the highlighted part on Page 17-18, line 914-950)

3. Policy level: increasing assessment and strengthening comprehensive strategies

The Region of the Americas is likely to show a lower prevalence of problematic physical fighting among adolescents than other regions. This study found that within the region, the prevalence in Paraguay is likely to be in the average or lower groups. Considering interpersonal violence as a leading cause of deaths and DALYs in the country, other sorts of interpersonal violence including bullying, emotional abuse, sexual and physical assault, and homicide as well as their contexts should be assessed so that comprehensive strategies and measures could be introduced to protect Paraguayan adolescents from problematic violence. (Kindly refer to the highlighted part on Page 19, line 992-996)

***

The summary which was added in the Conclusion section: (Page 20, line 1051-1061)

Considering serious interpersonal violence is causing deaths among Paraguayan adolescents, other kinds of violence including bullying, emotional abuse, sexual and physical assault, and homicide, as well as their backgrounds, should be assessed in order that comprehensive strategies and measures will be introduced to protect Paraguayan adolescents from problematic violence. Furthermore, in addition to the good progress in the implementation and enforcement of laws to advance the prevention of violence, it is be critical to strengthen social norms and values against violence as well as practical responses such as providing mental health services for both victims and perpetrators. To support the prevention of violence, the bans on corporal punishment in schools and at home and the changes in the relationship between parents and children should be strongly promoted by tireless awareness raising and the practical executions of the legislation.

**Conclusion:**

**1.** Lines 354-355 – Useful how? Please rephrase to say how the findings may be useful to inform policy and planning. What intervention programs are in place as best practice in Paraguay or other countries/ what is recommended in other countries?

We added the summary described above in the Conclusion section. (Page 20, line 1051-1061)
