# Supplementary material for: Prevalence and correlates of physical fighting among adolescents in Paraguay: Findings from the 2017 national school-based health survey
Source: PLoS One. 2022 Dec 30;17(12):e0279402. doi: 10.1371/journal.pone.0279402 (PMC9803110; doi:10.1371/journal.pone.0279402)
Supplement: Attachment — Submitted filename: Response to Reviewers 20220826.docx [file pone.0279402.s007.docx]

Manuscript ID: PONE-D-21-03008

Response to Reviewers

To

Dr José Antonio Ortega

Academic Editor

PLOS ONE

Dear Dr José Antonio Ortega

Thank you very much for your consideration of our manuscript, Physical fighting and associated factors among adolescents in Paraguay the 2017 national school-based health survey. We have revised the manuscript thoroughly according to the comments by the editor. We addressed all points clearly in the revised manuscript.

Our responses to the editor are provided as below. Kindly find our response in blue per each point.

Also, we have revised the manuscript and the corresponding sentences in the texts have been highlighted using track changes. All line numbers referred to in our responses below are in the margins of our revised manuscript with tracked changes.

We hope that the revisions are satisfactory in addressing issues raised by the editor and look forward to hearing your decision about this article.

Yours sincerely,

Authors

**=======================================================**

There has been a change in the academic editor since the previous draft. The two referees were available for comments indicating that their suggested changes had been adopted.

Thank you very much for sharing these.

There is still a need for an important copy-edit. Many sentences do not make grammatical sense or are clumsily written. I give some instances, but you should carry out a thorough revision with a native English speaker and/or grammatical software such as Grammarly.com.

Thank you for your suggestion. The manuscript was re-edited by an English-language editor and, in addition, was double-checked and updated using Grammarly.com.

Overall, it is felt that reporting is not complete, in particular regarding the model formulation. PLOS ONE endorses the use of the STROBE checklist, www.strobe-statement.org, to ensure thorough reporting.

We filled in the STROBE checklist and will include it in the submission. Could you find it for your reference?

The rationale of the study design is not clear: the use of the 2-fights.

- Why to focus on 2 or more fights? Why not dealing with the original variable with an ordered-logit, for instance?

We classified fighting behaviour into what could be considered potentially problematic (which has the possibility to result in short or long term sequalae) and potentially non-problematic (rough play). This categorization has been taken into consideration and now well understood from the psychosocial aspects of adolescent development and behaviour. For example, rough play should not in and of itself be classified alongside problematic behavioural patterns. It is instead characteristic of rapid bio-psychological, psycho-social and environmental adjustment as adolescents navigate their way into adulthood.

The aim with the above classification lies in avoiding pathologizing adolescent development patterns, particularly the behaviour of adolescent males who may display higher levels of physical aggression, but this aggression does not in and of itself constitute problematic behaviour.

Studies on adolescent aggression demonstrate that there is a difference between aggression and roughness among adolescents. Roughness during play may be associated with aspects of peer affiliation, although those on the other side of the aggression may need to defend themselves (1). Rough play is when a fight occurs without demanding submissiveness and distress to the victim (1). On the other hand, aggressiveness goes beyond roughness when the aggressor is likely to persist until they achieve a level of submissiveness from the victim (2). In this regard, the intention is to achieve dominance over peers and is likely to target potential victims who appear weaker in order to defeat them, and achieve a higher dominance reflected by the number of victories (2). Engagement in roughness and escalation to aggression with other peers is done to demonstrate dominance by taking on various fights (2).

In relation to the present study, the justification for cut-off in fights occurring at least 2 times or more was to include adolescents who may have been involved in problematic aggressive behaviour. Thus, the problematic behaviour is when one person is considered to have been involved in fights several times, either as the aggressor or the individual on the receiving end of the fighting behaviour. The non-problematic behaviour was considered to have occurred when one was not involved in fights at all or only once during the 12 months preceding the survey. This is because participation in one fight may not have been with the intention to achieve dominance but rather the fight could have been a random occurrence or a one-off disagreement with a peer. The challenge is that data on the reason for participating in the fights was not available and setting the cut-off at this level was to distinguish between frequent fighting and no or non-frequent fighting.

We described the point in the manuscript. (Line 187-192)

The rationale of the study design is not clear: the selection of variables.

The variables were selected based on previous studies which studied the physical fighting among school-attending adolescents using GSHS data from various other countries as cited in reference numbers (3, 11-16, 22-26). (Line 169-170)

The choice of variables in the final logistic regression model were based on the results of bivariate analyses. (Line 226-228)

- Line 39-40: Remove the second “only”

Thank you. We deleted it. (Line 39)

- L. 90: Experienced physical attacks, or “were physically attacked”. Not “experienced 91 physically attacked”.

We discussed and revised as “having been physically attacked” to keep the interviewees’ passive experiences in the past. (Line 99)

- Again in l. 94. In general, I’d suggest to use “physical attack” instead of “physically attacked” which is not grammarly. Eg: lowest prevalence of physical attack

We discussed and revised as “having been physically attacked” to keep the interviewees’ passive experiences in the past. (Line 100-101)

- L. 96: Should be “the same method of analysis”

We revised it. (Line 104-105)

- Line 114-116: You should give references and mention what they found regarding the correlates of physical fighting. The variables are coming out of the blue now and you are not explaining the rationale for their inclusion. You are mentioning some of these aspects in the discussion. This is OK. But these studies should have been mentioned and summarized in the introduction so that the reader knows what to expect and why.

Thank you for your suggestion. We added the summarised findings from the prior studies regarding the association between physical fighting among school-attending adolescents and family- and peer-level factors. (Line 107-109)

- L. 120-128: Try to improve the writing. Eg: Avoid reiteration of "use", of "specified".

We revised the paragraph (Line 139-140) as well as the others in the whole manuscript.

- L. 126, should be “in” the second stage,

We revised it as suggested. (Line 141)

- You do not mention the use of sampling weights in the analysis. You should use them given the 2-stage design to ensure that the analysis is statistically representative. (You mention weighting the proportions in line 186, but then table 2 is contradictorily not weighted. You also should use and mention weights in the logistic regression).

The last paragraph/sentence in the ‘Statistical analysis’ section is revised to clarify the analysis approach used, as under: (Line 232-235)

“Design-based analyses were carried out using Stata 16 program (StataCorp, 2019), by taking into account the complex survey design. All proportions, results of chi-square tests, and logistic regression models are reported based on design-based analysis; while unweighted counts/frequencies are reported.”

In table 2, the square brackets show the unweighted counts i.e. frequencies, while all the reported percentages are weighted; the results of tests and their p-values are all based on the statistical tests which accounted for the complex survey design.

Table 3 shows the results of design-based logistic regression models which took into account the complex survey design.

We also added these points to the tables. (Line 264-267; Line 291)

- Interesting that El Salvador is mentioned as having low prevalence of physical attack despite having the highest homicide rates in the world in 2019 (https://ourworldindata.org/homicides) and the highest proportion of deaths from homicides. What could be going on? Eg: One possible caveat of a retrospective study such as this is the possible lethality of the physical attacks. The survivors will have less attacks. Another possibility is underreporting due to fear of possible implications of declaring an attack. A third, sample selection of the less violent, the more violent being out of school. All of these factors could be mentioned as a limitation of this type of study.

These are indeed important points. We added two points in the limitation.

Students who were physically attacked to the point of death were not included in the survey (survivorship bias). (Line )

Third, this study used a self-report survey. This might have introduced a social-desirability bias and a relevant bias to the study results. It includes fears of possible implications of declaring attacks. (Line 459-460, 464-465)

Reference

1.	Neill SRSJ. AGGRESSIVE AND NON-AGGRESSIVE FIGHTING IN TWELVE-TO-THIRTEEN YEAR OLD PRE-ADOLESCENT BOYS. Journal of Child Psychology and Psychiatry. 1976;17(3):213-20.

2.	Pellegrini AD. A longitudinal study of boys' rough-and-tumble play and dominance during early adolescence. Journal of Applied Developmental Psychology. 1995;16(1):77-93.
