# Supplementary material for: Prevalence and correlates of physical fighting among adolescents in Paraguay: Findings from the 2017 national school-based health survey
Source: PLoS One. 2022 Dec 30;17(12):e0279402. doi: 10.1371/journal.pone.0279402 (PMC9803110; doi:10.1371/journal.pone.0279402)
Supplement: Attachment — Submitted filename: Response to Reviewers 20221101.docx [file pone.0279402.s008.docx]

Manuscript ID: PONE-D-21-03008

Response to Reviewers

To

Dr José Antonio Ortega

Academic Editor

PLOS ONE

Dear Dr José Antonio Ortega

Thank you very much for your consideration of our manuscript, Physical fighting and associated factors among adolescents in Paraguay the 2017 national school-based health survey. We have revised the manuscript thoroughly according to your comments. We addressed all points clearly in the revised manuscript.

Our responses are provided as below. Kindly find our response in blue per each point.

Also, we have revised the manuscript and the corresponding sentences in the texts have been highlighted using track changes. All line numbers referred to in our responses below are in the margins of our revised manuscript with tracked changes.

We hope that the revisions are satisfactory in addressing issues and look forward to hearing your decision about this article.

Yours sincerely,

Authors

**=======================================================**

Thank you for submitting your manuscript to PLOS ONE. After careful consideration, we feel that it has merit but does not fully meet PLOS ONE’s publication criteria as it currently stands. Therefore, we invite you to submit a revised version of the manuscript that addresses the points raised during the review process.

It is felt that the manuscript has substantially improved. A few minor changes, mostly typos, emerge from the reading.

Thank you very much for your comments and careful review.

l. 101 Should be: were regarded **as** protective attributes

We revised the part as suggested. (Line 102)

Both in the abstract and in the main text you mention “3,149 students at Octavo-Tercer Curso completed the survey questionnaire”. This is not clearly understandable. It literally means in Spanish “eight-third course”. I assume it is eight year of primary to third year of secondary, but this is unclear. Why not refer in both instances, as described in the survey documentation “UNIVERSE School-going adolescents aged 13-17 years.”

In Abstract, the sentence has been revised as below:

This survey collects health-related information on school-attending adolescents aged 13-17 years. (Line 35)

And in Materials and Methods section as below:

In Paraguay, 3,149 students at the age of 13-17 years completed the survey questionnaire

(Line 138)

Line 145: You describe variable by variable missing information. It would be good to add a summary sentence saying: As a result a total of ---- observation were dropped due to missing information on some variable”. Note that several variables can be missing. If my reading is incorrect and each sentence refers to additional missing cases, then rephrase and make it clear.

Thank you for this comment. Owing to missing information in various covariates, the total number of observations/records used in the final multivariable logistic model were 2,520. We added the following sentence to further clarify.

“As a result, a total of 2,520 observations were used in the final multivariable logistic regression model, for information was available for all variables.” (Line 253-254)

L. 264: should be “the two most protective associations were the attributes of having helpful peers and supportive parents”

We revised the part as suggested. (Line 269-273)

L. 270: Counties should be countries

We revised the part as suggested. (Line 278)

L 361: Multicountry, not multicounty

We revised the part as suggested. (Line 369)

L. 378: to the point of death or dropout might be prefarrable. You are also missing children not at school.

Thank you for your suggestion. We agreed on it and added dropout in the sentence as underlined.

Regarding children not at school, we described children who are not at school due to various reason in the prior description and added another example as underlined.

“First, this is a cross-sectional study which captured only the responses of the students who attended school on the day of the survey. It could mean, for example, that although our study did not identify food deprivation as a prevalent attribute, students from impoverished households might not have been at school on that day or might not have been attending school at all. Students with only a few close friends might also have been unwilling to be at school. Students who tend to play truant from schools may not be included in the survey. Students who were physically attacked to the point of death or dropout were not included in the survey (survivorship bias).” (Line 380-387)
