# Supplementary material for: Prevalence and correlates of physical fighting among adolescents in Paraguay: Findings from the 2017 national school-based health survey
Source: PLoS One. 2022 Dec 30;17(12):e0279402. doi: 10.1371/journal.pone.0279402 (PMC9803110; doi:10.1371/journal.pone.0279402)
Supplement: Attachment — Submitted filename: Response to Reviewers 20221205.docx [file pone.0279402.s009.docx]

Manuscript ID: PONE-D-21-03008

Response to Reviewers

To

Dr José Antonio Ortega

Academic Editor

PLOS ONE

Dear Dr José Antonio Ortega

Thank you very much for your consideration of our manuscript, Physical fighting and associated factors among adolescents in Paraguay the 2017 national school-based health survey. We have revised the manuscript thoroughly according to your comments. We addressed all points clearly in the revised manuscript.

Our responses are provided as below. Kindly find our response in blue per each point.

Also, we have revised the manuscript and the corresponding sentences in the texts have been highlighted using track changes. All line numbers referred to in our responses below are in the margins of our revised manuscript with tracked changes.

We hope that the revisions are satisfactory in addressing issues and look forward to hearing your decision about this article.

Yours sincerely,

Authors

**=======================================================**

Thank you for submitting your manuscript to PLOS ONE. After careful consideration, we feel that it has merit but does not fully meet PLOS ONE’s publication criteria as it currently stands. Therefore, we invite you to submit a revised version of the manuscript that addresses the points raised during the review process.

Thank you very much for your comments and careful review.

The article is basically ready for publication, except for the quality of the English that still requires improvement. In particular the new edited paragraphs do not read well. Also, maybe you are not interpreting correctly the concern on not covering the complete population. It is a concern only to the extent that this is implies selection on your dependent variable, physical fighting. Also some judgmental expressions like playing truant are better to be avoided. Also you acknowledged that the design does not allow causal statements, but the choice of variables also does not help. Eg: could not attacks be a result of previous fights and not otherwise?

Please replace

First, this is a cross-sectional study which captured only the 375 responses of the students who attended school on the day of the survey. It could mean, for 376 example, that although our study did not identify food deprivation as a prevalent attribute, 377 students from impoverished households might not have been at school on that day or might 378 not have been attending school at all. Students with only a few close friends might also have 379 been unwilling to be at school. Students who tend to play truant from schools may not be 380 included in the survey. Students who were physically attacked to the point of death or dropout 381 were not included in the survey (survivorship bias). Second, cross-sectional data do not confer 382 causal relationships between attributes studied.

With

First, this is a cross-sectional study which captured only the responses of the students who attended school on the day of the survey. School attendance could be correlated with the risk of physical fighting and being physically attacked due to sequels of previous episodes or preventive behaviour. Second, the cross-sectional nature of the data does not allow causal interpretation of the associations studied. Reverse causality could also affect behavioural variables such as being physically attacked.

389 among school-attending adolescents in Paraguay. We also used the most **updated** survey data

Change updated to **recent**

Thank you very much for your clarification and suggestions. We revised the parts as suggested. (Line 379-384, 405)

We also had another English check through the manuscript.

END
